# The use of ultrasound in the diagnosis of a ligated spinal accessory nerve with sural graft repair

**DOI:** 10.1016/j.radcr.2026.01.069

**Published:** 2026-02-27

**Authors:** Marion Hanley, Benjamin Wall, Simon Ferrero, Alex O’Beirne, Daren Gibson

**Affiliations:** a Radiology Department, Fiona Stanley Hospital, Perth, WA, Australia; b Orthopaedic Department, Fiona Stanley Hospital, Perth, WA, Australia

**Keywords:** Spinal accessory nerve, Cranial nerve 11, Greater auricular nerve, Stump neuroma, Ultrasound

## Abstract

The extra-cranial spinal accessory nerve (SAN) is an important motor nerve, responsible for innervating the trapezius and sternocleidomastoid muscle (SCM). At a particular point in the neck SAN is vulnerable to iatrogenic injuries, particularly during lymph neck node dissection. We present the radiological and clinical features of a 29 year old female sickle cell patient who was referred for an ultrasound assessment of a suspected injured SAN following an excisional lymph node biopsy. A sural graft repair was subsequently preformed of both SAN and the greater auricular nerve (GAN). This case report aims to promote the diagnostic capabilities of ultrasound in identifying stump neuromas, specially relating to the SAN.

## Introduction

Peripheral nerve injuries in the head and neck are often as a result of an iatrogenic injury and can range from mild neurapraxia to debilitating morbidity. The most common cause of peripheral nerve injury is trauma [1]. There are twelve cranial nerves, running outside the cranium to innervate vital structures. Injury to cranial nerve XI, also known as the spinal accessory nerve, can lead to a loss of motor function of the ipsilateral trapezius muscle, winging scapula, neuralgic pain and scapula-thoracic stiffness [2]. It is important to timely diagnose such an injury in order to optimize early intervention and reduce long term morbidity. We present the interesting case of iatrogenic injury to both the spinal accessory nerve and greater auricular nerve with a key diagnostic role of ultrasound (US). This case highlights the usefulness of diagnostic ultrasound in the assessment of peripheral nerve injuries, particularly to the SAN.

### Case report

A 29 year old female patient, with a background history of Sickle cell disease alpha thalassemia trait, presented with palpable bilateral cervical lymphadenopathy. Cervical lymph node fine needle biopsies demonstrated an inflammatory profile on histopathology. However, a diagnosis of tuberculosis was suspected and thus a cervical lymph node excision biopsy was sought. At the time of surgery, a left oblique lateral neck incision was made the over palpable coalescent lymph nodes. The lymph nodes were adherent to the surrounding structures with pus expressed on handling. The excision was challenging.

Immediately following surgery, the patient developed pain and significant loss of function of her left shoulder. On examination, there was total loss of shoulder abduction (Grade 0/5) and significant loss of contralateral neck rotation with flexion. The patient struggled with basic tasks around daily personal care. She also reported new paraesthesia her left earlobe and mandibular ramal area.

**Fig. 1:**
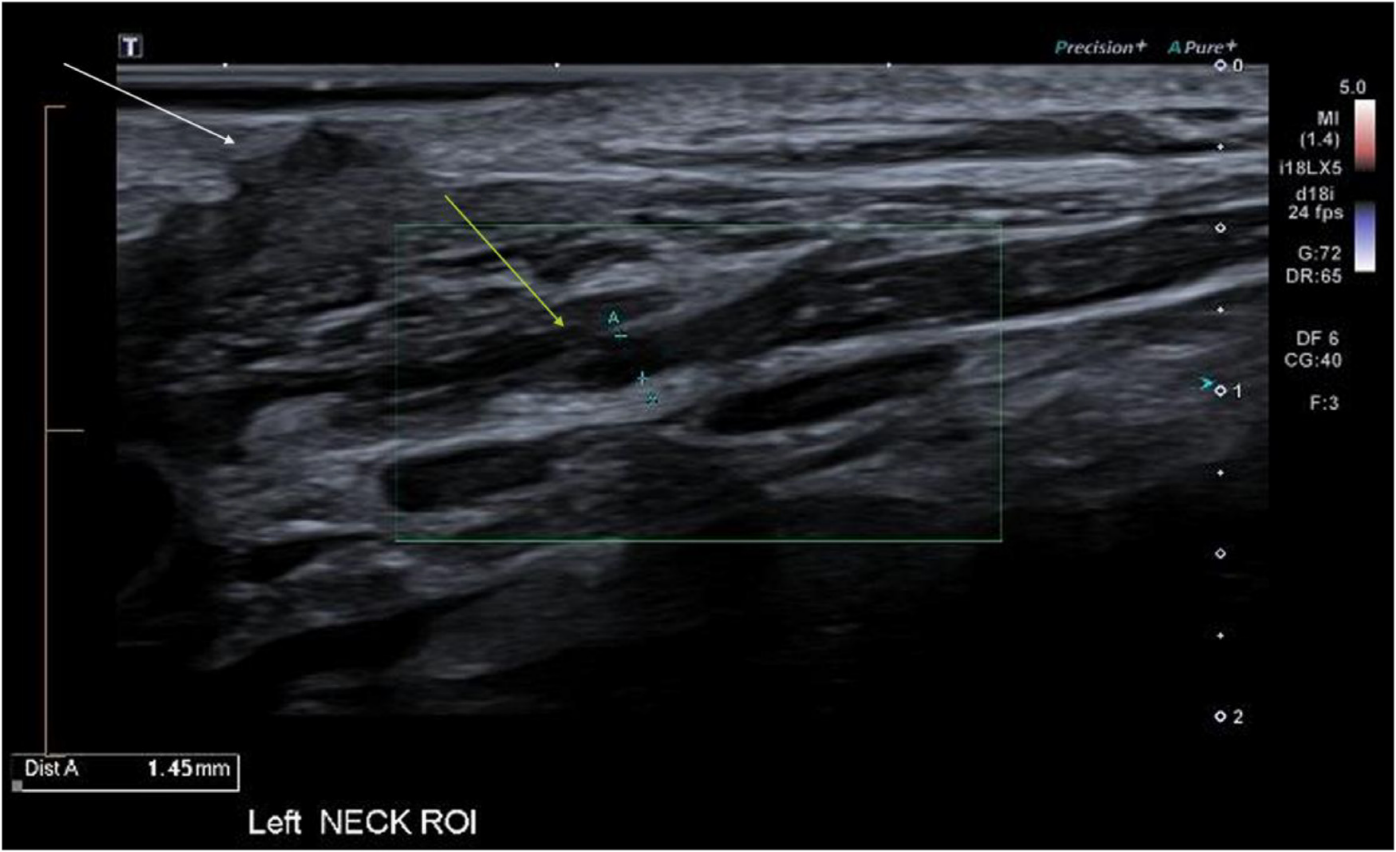
US image of the left spinal accessory nerve (SAN) stump neuroma, measuring 1.45mm in cross-section (yellow arrow). Distally, the nerve measures 0.7 mm. The white arrow indicating the skin incision near the ligated SAN.

**Fig. 2:**
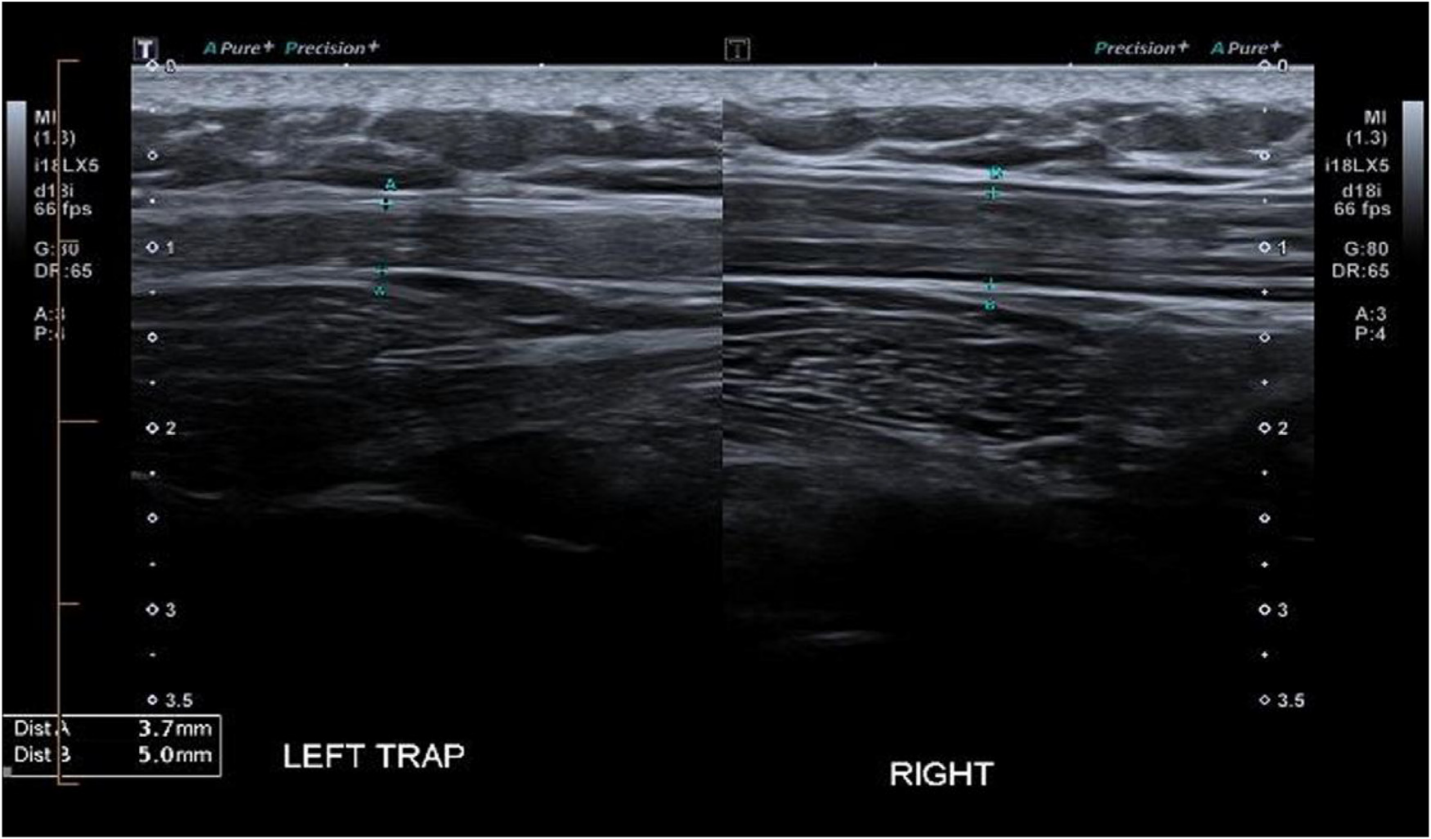
US images of the left trapezius muscle measuring 3.7mm in cross-section and comparatively with the right trapezius muscle measuring 5.0mm in cross section, in keeping with left sided muscular atrophy secondary to ligated left sided SAN.

**Fig. 3:**
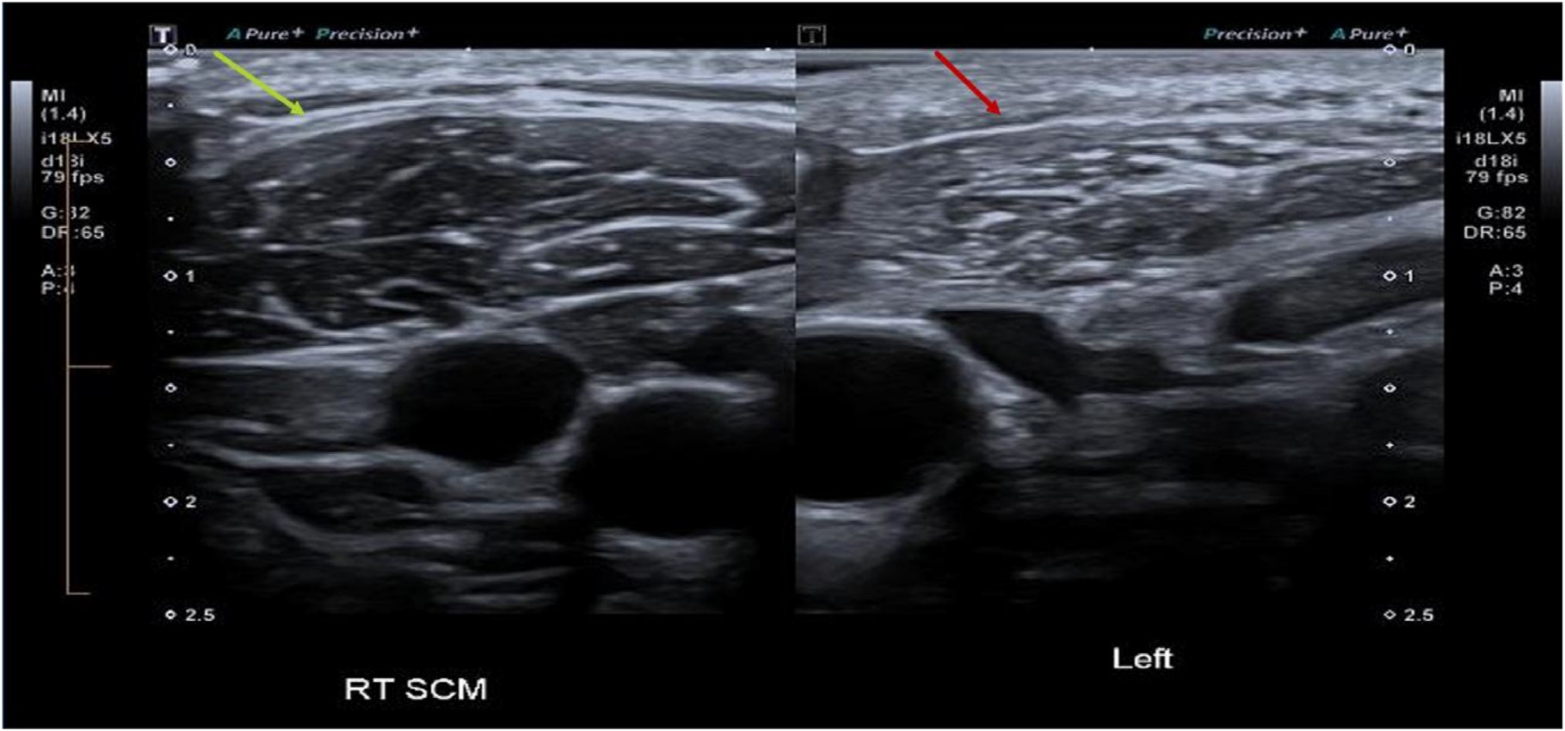
Ultrasound images demonstrating marked reduction in muscle volume and fatty infiltration of the left sternocleidomastoid muscle (SCM), red arrow, in comparison to the right SCM muscle, yellow arrow.

A focused ultrasound examination was requested to assess for evidence of nerve injury, specifically related to the SAN. The examination was focused on the left neck incision, over the posterior border of the SCM. Over this region a discontinuity of an enlarged nerve was identified. By assessing its relationship to the anterior and posterior triangle, the neuroma was identified as the SAN (Fig. 1). There was also early associated muscle denervation changes with mild fatty atrophy of left trapezius and SCM, most notable when a comparison was made with the contralateral non-atrophied right sided muscles (Fig. 2, Fig. 3).

The patient subsequently underwent electro-physiological nerve conduction studies confirming the ligated spinal accessory nerve and also of the GAN, with no electrical impulses detected. A decision was made three weeks following the initial insult to surgically repair the ligated SAN and GAN.

**Fig. 4:**
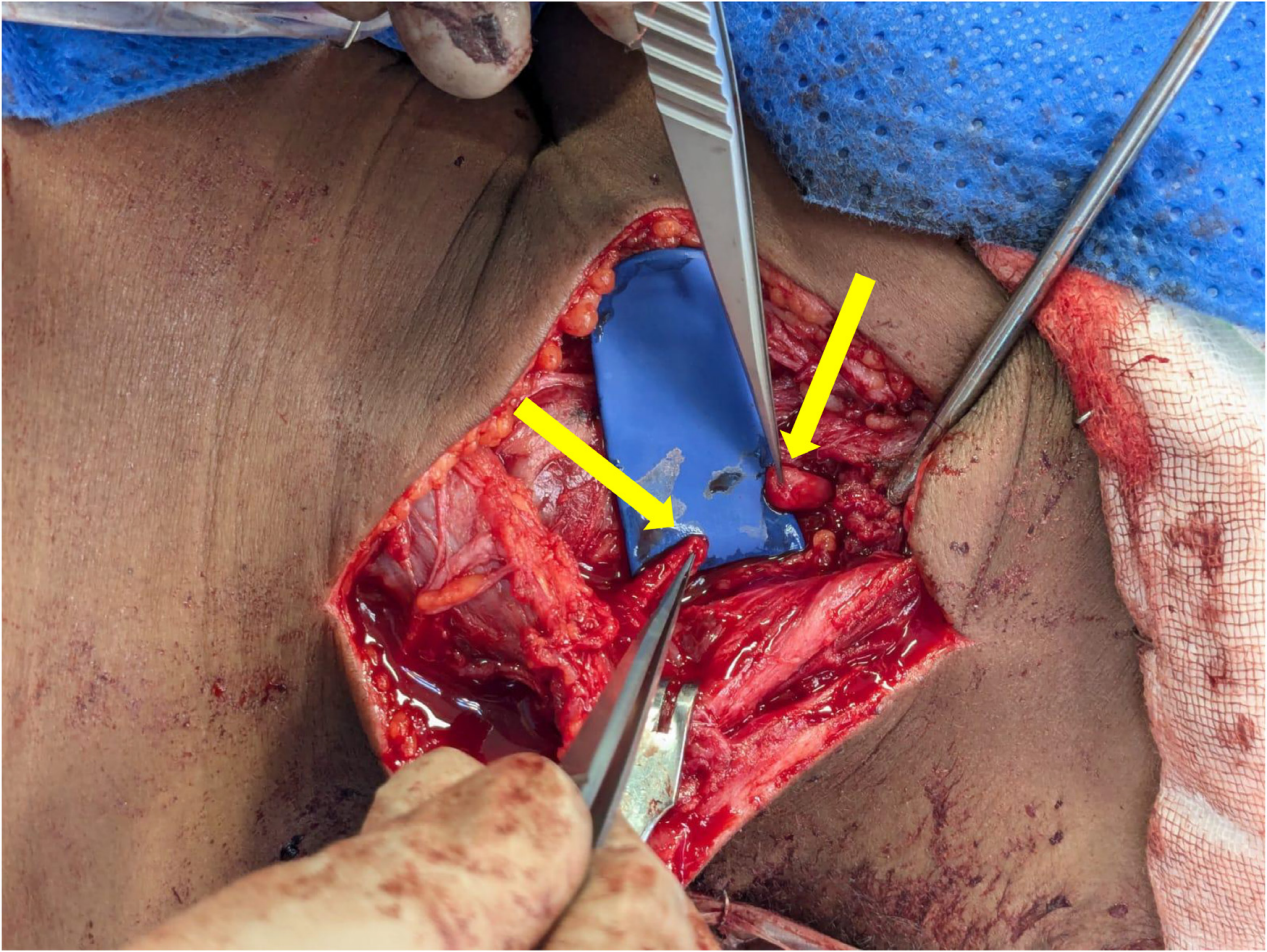
Intraoperative images demonstrating the two enlarged ends of the mobilized ligated SAN (yellow arrows).

**Fig. 5:**
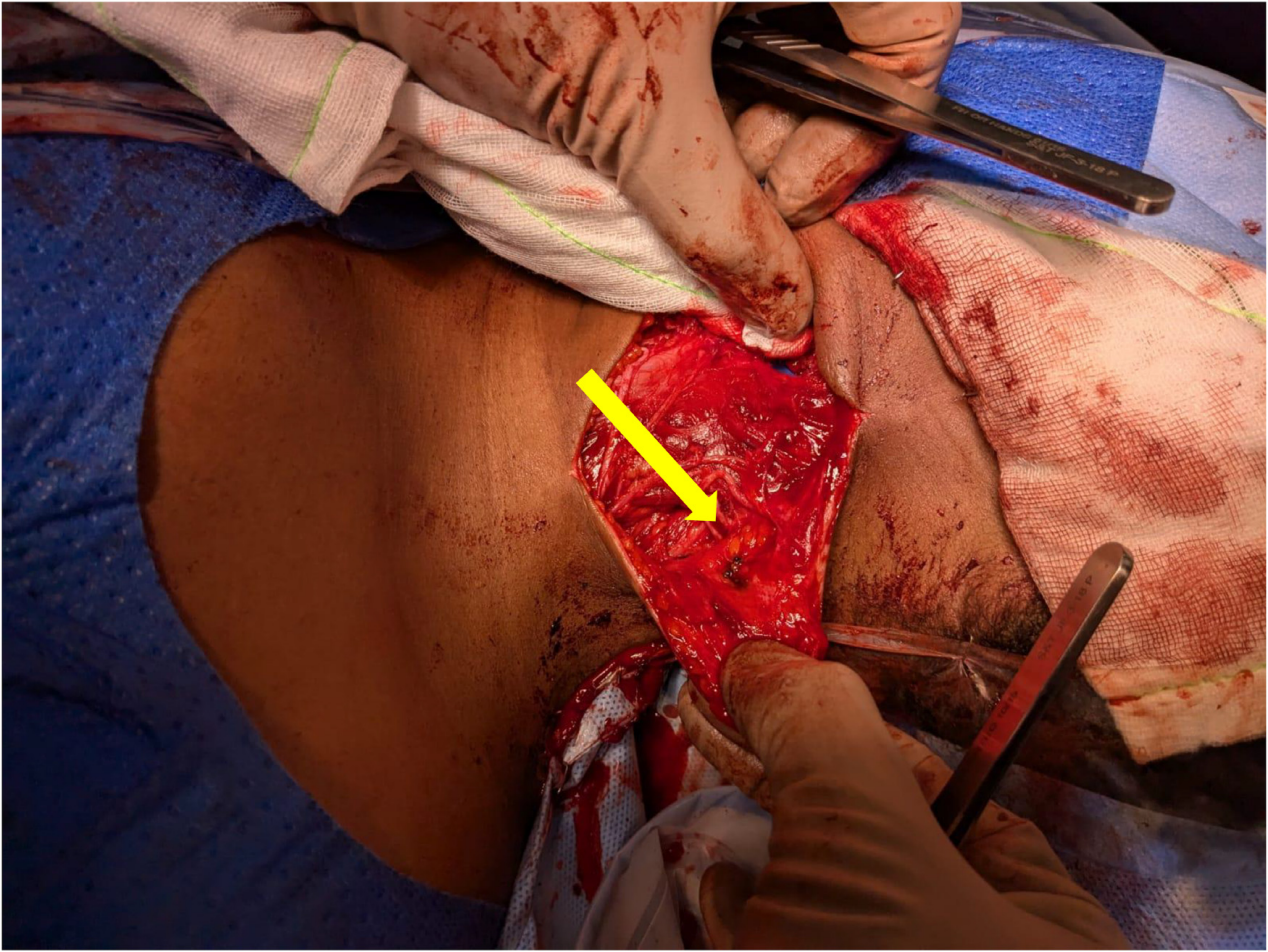
Intra-operative image. Completed repair with Remplir wrap around SAN (yellow arrow).

**Fig. 6:**
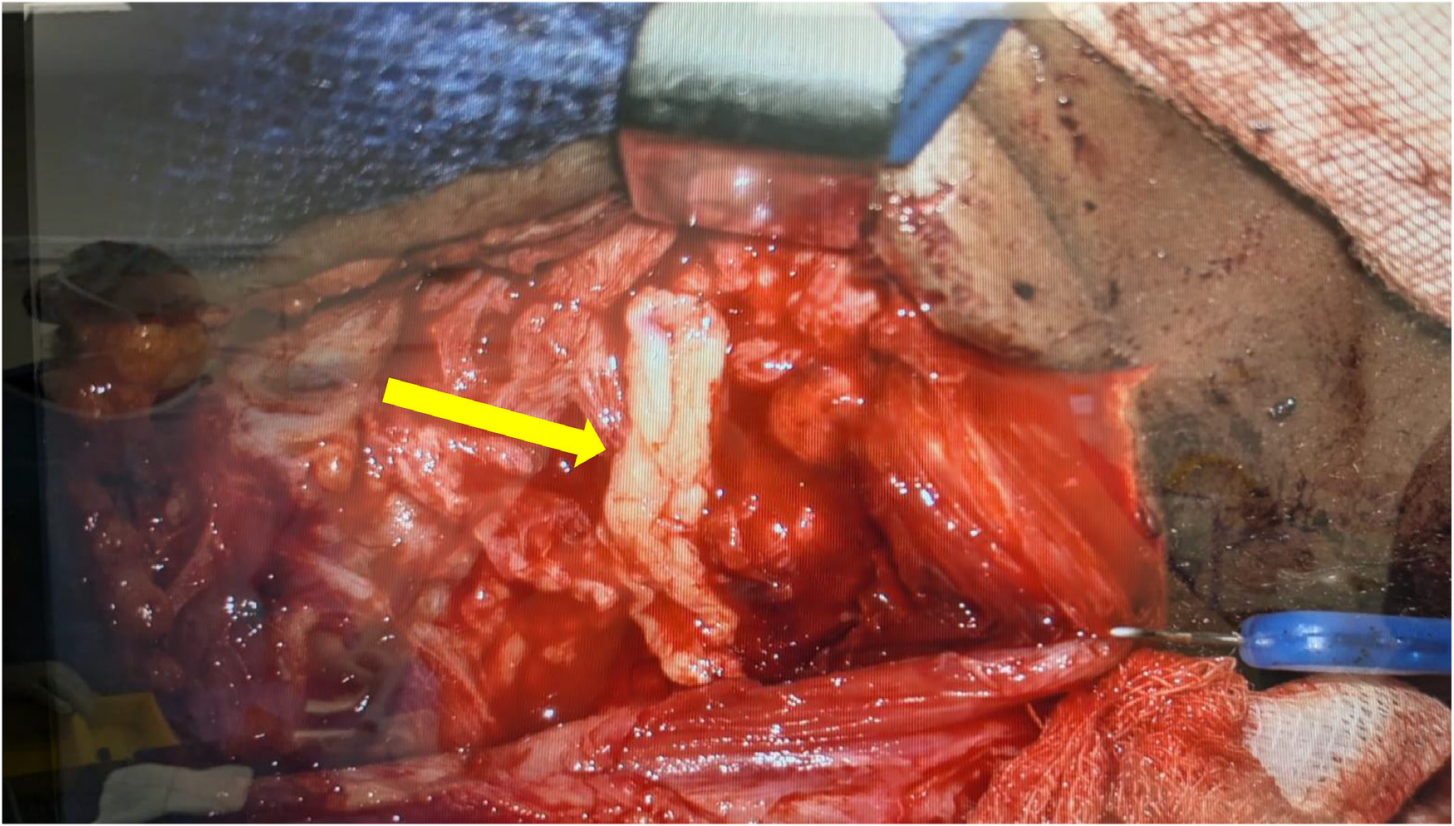
Intra-operative image. GAN with sural nerve graft (yellow arrow).

Surgical exploration confirmed the 3cm neural discontinuity of the ligated SAN (Fig. 4). The patient underwent a right sided sural nerve harvest in which was utilized for an interposition graft reconstruction of the SAN and GAN (Fig. 5, Fig. 6).

Our patient demonstrated technical surgical success with little morbidity at the graft site. 6 months post-surgery, the patient experienced neuropathic type pain in the distribution of the SAN and GAN that suggested early nerve recovery. There was also a significant improvement of trapezius power, Grade 3/5 at 6 months post-op. There was however, ongoing loss of motion. Trapezius muscle strength continued to improve with an intense physiotherapy rehabilitation programme, achieving Grade 4/5 power 1 year after the neural graft repair. The patient also reported marked improvement in sensation around the lower part of her jaw up into the malar region of her cheek, along the GAN distribution. It was advised to the patient that it would take two years for maximum recovery after nerve grafting.

## Discussion

The XI cranial nerve is composed of two parts, the cranial and the spinal components. The spinal part supplies motor innervation to the ipsilateral sternocleidomastoid and trapezius muscles. Should an injury occur to SAN, the loss of shoulder abduction can be devasting, leading patients unable to perform routine practices of daily living. Shoulder pain and stiffness can progress and become increasingly symptomatic when there is a delayed diagnosis and repair [2].

These injuries occur not infrequently during difficult, complicated neck lymph node excisional biopsies, with a reported frequency in the range of 3-8% [3]. In a large study of 111 patients who underwent surgical repair of SAN, 93% of injuries were iatrogenic and 80% of those injuries involved lymph node biopsies [2]. A specific vulnerable junction is located at the posterior border of the sternocleidomastoid muscle; where the nerve transverses between the anterior and posterior triangles.

**Fig. 7:**
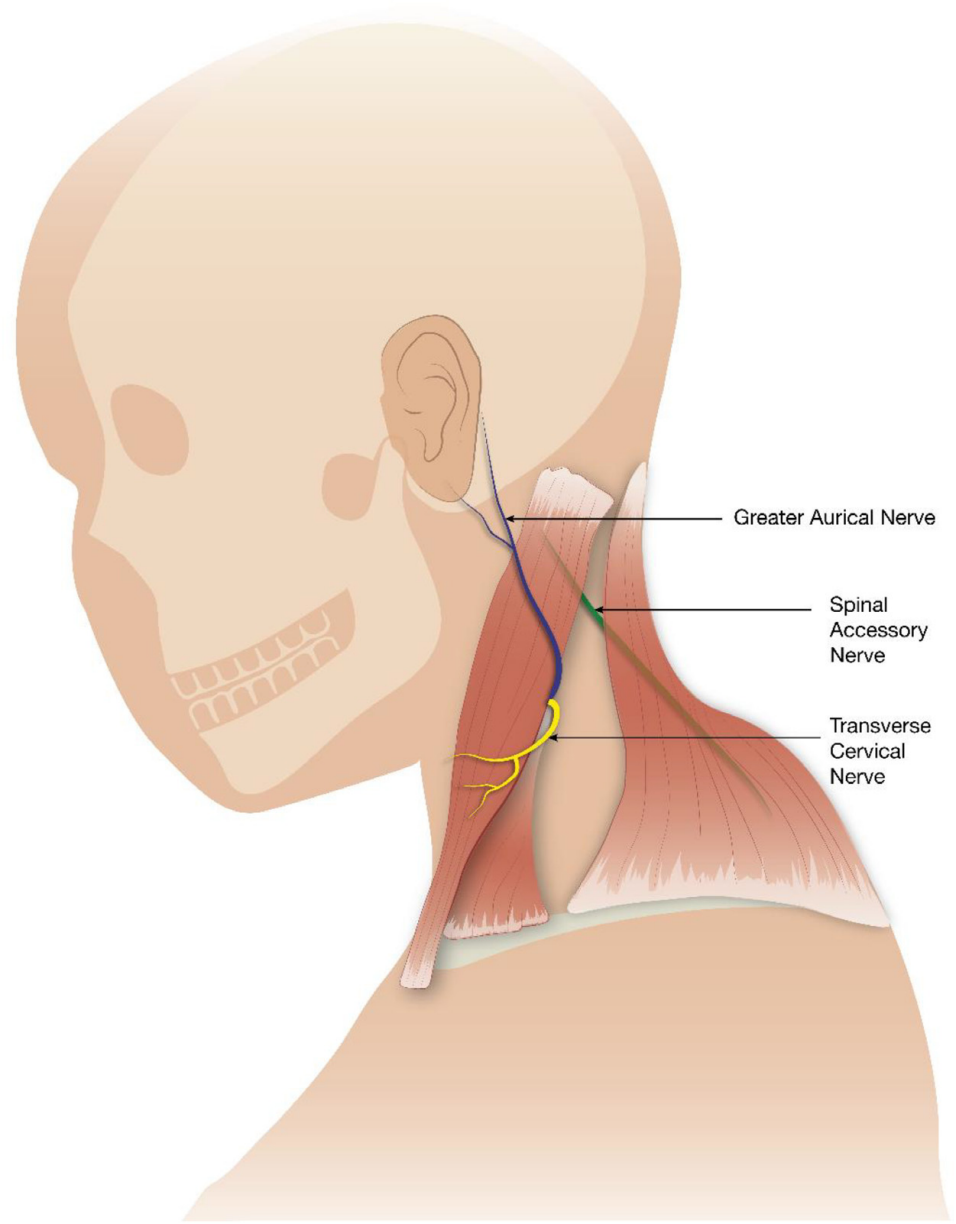
Sagittal illustration of the course of the SAN, GAN and TCN.

**Fig. 8:**
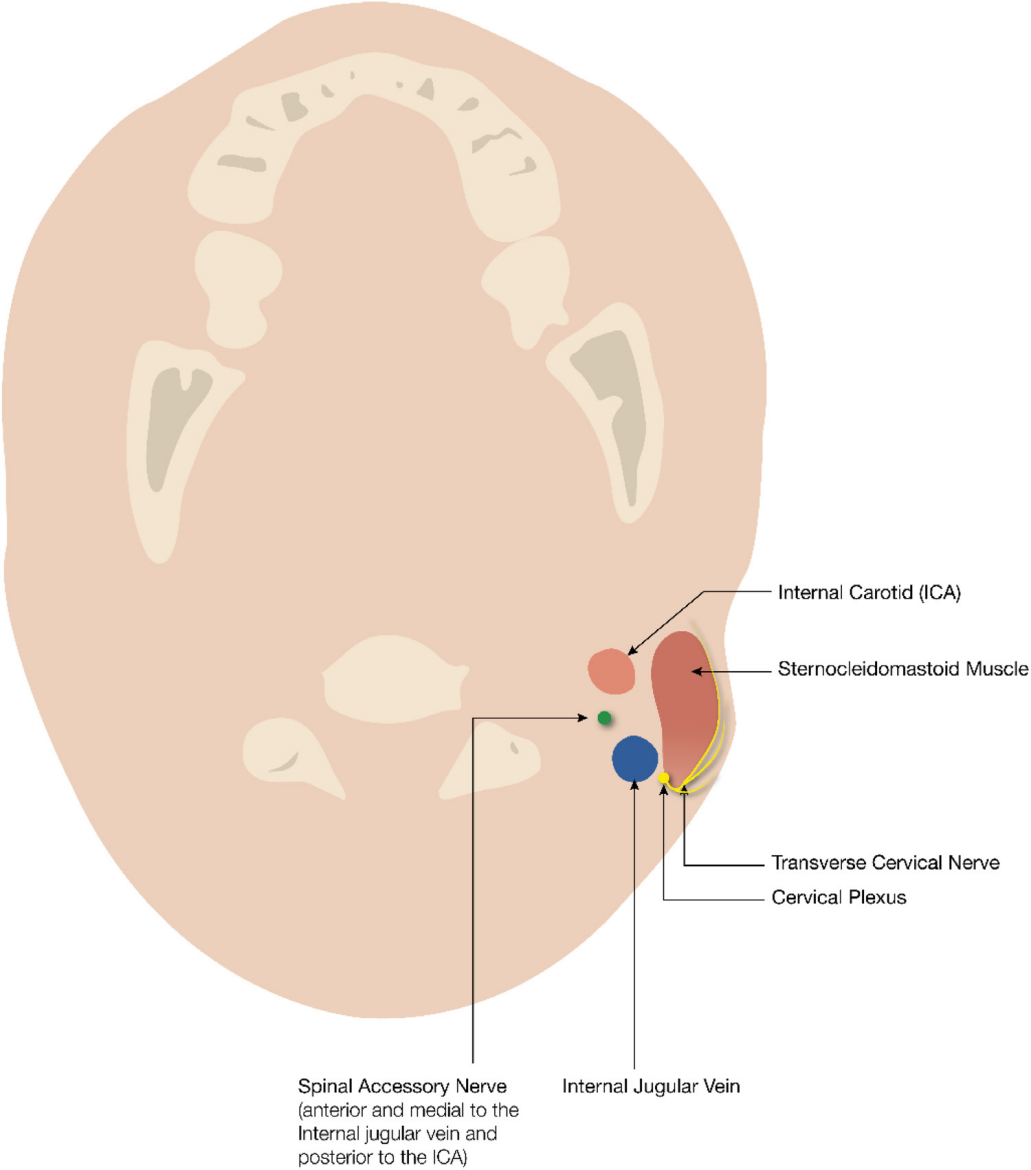
Axial illustration of the relationships between the SAN and the internal jugular vein and internal carotid artery at the level of the jugular foramen.

The variable relationship between SAN and GAN is an important one anatomically. GAN is a superficial part of the cervical plexus (C2-C3). It provides sensory innervation to the skin of the auricle, the skin overlying the parotid gland and mastoid process. GAN invariably courses along the deep posterior border of the sternocleidomastoid muscle. The emerging SAN can usually be identified 10 mm superior to this point [4] (Fig. 7, Fig. 8). This special neural geographical relationship aids in the identification of the spinal accessory nerve in an attempt to avoid injury during head and neck procedures.

The role of US in the assessment of peripheral nerves is well documented as a cost-effective and helpful tool [5,6]. Its sensitivity rate at detecting peripheral nerve injuries is as high as 93% [6]. Traditionally electromagnetic nerve conduction studies are used to assess for peripheral nerve injuries, however they are uncomfortable for patients and are not as accurate in the setting of an acute nerve injury [6]. Ultrasound has been shown to modify the diagnosis and treatment in 58% of patients with traumatic nerve lesions when added to standard neurophysiologic assessment [6].

While there is limited literature on US in assessing SAN injuries, a recent study showed 100% visibility in SAN assessment in 60 healthy volunteers [7]. This study also showed that ultrasound correctly identified injuries to SAN, confirmed thereafter with MRI or surgery [7]. The anatomical land marks described above can be used with confidence to locate the SAN.

Secondary signs of SAN injury including fatty muscle atrophy can also be readily visualized with US, highlighting another diagnostic component of US. Our case also adds to the growing body of evidence that the simple, non-ionizing tool of US can be used to help diagnose SAN injuries.

Pre-operative ultrasound can also identify the SAN in the posterior triangle, although this is not routine practice [8].

Treatment options for spinal accessory nerve injuries include; neurolysis, direct nerve repair and nerve grafting, all of which depends on the nerve continuity. A neuroma in discontinuity is usually treated with a graft repair. The early diagnosis enabled the patient described in this case study to be correctly treated and managed. EMG studies may not be as beneficial in such an acute setting and thus US facilitated the streamlined urgent surgical referral, once a neuroma was accurately diagnosed.

Repair of the SAN is challenging. In one large study of 111 surgically repaired SANs, 58 underwent nerve graft repair with either a local nerve (*n* = 33) or a sural nerve repair (*n* = 25). There were no post-operative differences in outcomes between those the two types of grafts used [2].

This same study described, reported 72% of patients had Grade 3 power post graft repair. This is comparable with other studies [9,10]. A larger study demonstrated that 85% of patients who underwent nerve graft repair demonstrated Grade 3 or higher improvement in the functional grading system described by Louisiana State University Health Sciences [10]. Our patient demonstrated technical surgical success with little morbidity at the graft site. Function improvement to Grade 4 occurred 1 year post repair with also improved sensation along the GAN distribution.

## Conclusion

Timely, early identification and specialist referral of SAN injuries is an important clinical and radiological diagnosis to make with nerve graft repair as a viable and successful treatment option. The role of US in assessing SAN injuries is important and probably underutilized in the radiology world. This case adds to the growing body of evidence promoting US to asses for injuries to SAN.

## Patient consent

Informed consent was obtained by the patient to proceed with this case report.
